# The Ins and Outs of Endosteal Niche Disruption in the Bone Marrow: Relevance for Myeloma Oncogenesis

**DOI:** 10.3390/biology12070990

**Published:** 2023-07-12

**Authors:** Jean-Pascal Capp, Régis Bataille

**Affiliations:** 1 Toulouse Biotechnology Institute, INSA/University of Toulouse, CNRS, INRAE, 31077 Toulouse, France; 2 School of Medicine, University of Angers, 49045 Angers, France

**Keywords:** multiple myeloma, MGUS, Gaucher disease, osteoblasts, osteoclasts, bone microenvironment, endosteal niche disruption, TiDiS theory

## Abstract

**Simple Summary:**

Monoclonal gammopathy cases of unknown significance occur mainly in aging people. Furthermore, it is now well-established that all cases of multiple myeloma, an aggressive lethal cancer of the bone marrow leading to bone destruction, emerge from such cases of gammopathy. In order to improve the management of myeloma, it is therefore critical to discover all of the specific factors responsible for their emergence. Here, we suggest that bone factors, i.e., bone senescence and bone inflammaging, could facilitate the emergence of these pathologies in aging people. The generalized bone loss observed in gammopathy and myeloma corresponds at the histological level to a disruption of the endosteal niche, which is both the site of hematopoiesis and the bone remodeling compartment and consists of a coupling between bone-forming osteoblasts and bone-resorbing osteoclasts. This niche undergoes a shift from an osteoblastic to an osteoclastic profile as soon as the physiological bone experiences senescence. Such a disrupted endosteal niche could represent the permissive microenvironment necessary for gammopathy and myeloma. Inside this environment, osteoclasts, through their capacity to suppress T cells but also present antigens in the bone marrow, could locally create immune changes favorable to the maintenance of gammopathy and its malignant transformation by favoring cellular instability.

**Abstract:**

Multiple Myeloma (MM) and its preexisting stage, termed Monoclonal Gammopathy of Undetermined Significance (MGUS), have long been considered mainly as genomic diseases. However, the bone changes observed in both conditions have led to a reassessment of the role of the bone microenvironment, mainly the endosteal niche in their genesis. Here, we consider the disruption of the endosteal niche in the bone marrow, that is, the shift of the endosteal niche from an osteoblastic to an osteoclastic profile produced by bone senescence and inflammaging, as the key element. Thus, this disrupted endosteal niche is proposed to represent the permissive microenvironment necessary not only for the emergence of MM from MGUS but also for the emergence and maintenance of MGUS. Moreover, the excess of osteoclasts would favor the presentation of antigens (Ag) into the endosteal niche because osteoclasts are Ag-presenting cells. As such, they could significantly stimulate the presentation of some specific Ag and the clonal expansion of the stimulated cells as well as favor the expansion of such selected clones because osteoclasts are immunosuppressive. We also discuss this scenario in the Gaucher disease, in which the high incidence of MGUS and MM makes it a good model both at the bone level and the immunological level. Finally, we envisage that this endosteal niche disruption would increase the stochasticity (epigenetic and genetic instability) in the selected clones, according to our Tissue Disruption-induced cell Stochasticity (TiDiS) theory.

## 1. Introduction

All Multiple Myeloma (MM) cases emerge from a pre-existing stage termed Monoclonal Gammopathy of Undetermined Significance (MGUS), confirming the ‘2-hits’ hypothesis presented by SE Salmon and M Seligman 50 years ago [1]. Actually, all MGUS cases already have the capacity for malignant transformation into MM. Indeed, because the malignant transformation rate of MGUS into MM is 1.3% per year without any plateau, it means that theoretically, with time, all MGUS cases could evolve toward MM [2]. Only the advanced age (>40 years) of individuals with MGUS can hamper this deleterious evolution. For a better understanding of the emergence of MM and to prevent this time-dependent capacity of MGUS cases to transform into those of MM, it appears critical to find out the contexts favoring the emergence of MGUS cases and their potential transition to MM, that is, the ‘permissive bone microenvironment’ to MGUS and MM.

**Figure 1:**
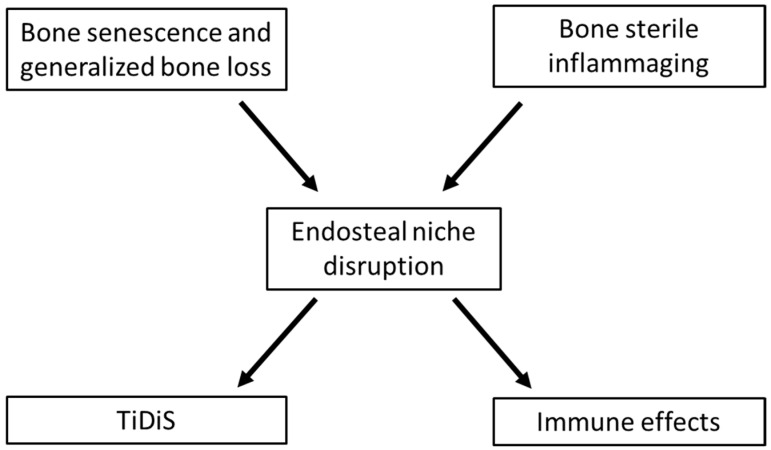
The ins and outs of endosteal niche disruption in the bone marrow: relevance for myeloma oncogenesis. The disruption of the endosteal niche in the bone marrow is primarily caused by bone senescence and inflammaging. It mainly consists of a shift from an osteoblastic to an osteoclastic profile. It is proposed that it represents the permissive microenvironment necessary for the emergence of both MGUS and MM. As osteoclasts are antigen-presenting cells, their excess is expected to stimulate the presentation of some specific Ag and the clonal expansion of the stimulated cells as well as to favor the expansion of the selected clones because they are immunosuppressive. This immunosuppression occurs through the increased activation of the programmed cell death ligand 1 (PD-L1), which, through engagement with PD-1 on activated T cells, leads to impaired T cell proliferation and cytotoxicity against MM cells (dysfunction, exhaustion, neutralization, and production of IL-10) [11]. The endosteal niche disruption would also increase the stochasticity (epigenetic and genetic instability) in the selected clones, according to the Tissue Disruption-induced cell Stochasticity (TiDiS) theory.

Together with MGUS, MM-induced bone changes (lytic bone lesions that are characteristic of MM) are the second major component of MM oncogenesis [3]. In addition to lytic bone lesions, MM-induced bone changes also include generalized bone loss [4]. Of note, this phenomenon is also observed in MGUS (reviewed in [5]). The histological bases of this generalized bone loss is a disruption of the endosteal niche, which corresponds to the bone remodeling compartment and is one of the two distinct microenvironmental niches within the bone marrow with the vascular niche where angiogenesis occurs. (Nevertheless, the dichotomy of the osteoblastic and vascular niches seems to be non-absolute, and the reconciling concept of endosteovascular niches has emerged [6,7,8]). Among these two niches, the endosteal niche disruption (END) is the most documented and involved in MGUS and MM oncogenesis. This suggests a common causal factor in continuation with the excessive bone senescence processes observed during aging, which is also characterized by such bone loss. In this review, we propose that the disruption of the endosteal niche in the bone marrow plays a pivotal role for both (i) the emergence (and maintenance) of MGUS cases and (ii) their malignant transformation into MM. In our previous works, we hypothesized the role of the END to induce/increase the stochasticity of the malignant clone in the frame of the Tissue Disruption-induced cell Stochasticity (TiDiS) theory [9,10]. Here, we progress further by considering the early events leading to this disruption (the ins: generalized bone loss in aging and inflammaging) and its oncogenic and immunological consequences (the outs: TiDiS and immune effects) (Figure 1).

Indeed, END, as a direct consequence of bone senescence and as abnormally augmented/accentuated by sterile bone inflammaging, could represent the permissive micro-environment necessary for the emergence of MGUS cases, their maintenance, and their subsequent malignant transformation into MM. Thus, we suggest that the END plays a critical role in the emergence of MGUS preceding their malignant transformation, and that this explains some immunological observations in both MGUS and MM. This scenario could also explain the high incidence of both MGUS and MM observed in Gaucher Disease (GD) cases [12], where bone disease seems to present the same mechanisms than in MGUS/MM and which is, thus, a good model both at the bone level and the immunological level.

## 2. Ins: Bone Senescence and Generalized Bone Loss in Aging

Bone cells are composed of osteoblasts (OB), their descendant osteocytes and osteoclasts (OC). Bone homeostasis is maintained through persistent remodeling mediated by bone-forming OB and bone-resorbing OC. Under normal conditions, the resorption of old bone is compensated by an equal amount of new bone formation, with a coupling between the two processes that should ensure the maintenance of stable bone mass. Any event inducing an imbalance between these processes by increasing bone resorption or decreasing bone formation, or both, can result in generalized bone loss, as observed in aging and osteoporosis. Indeed, bone architecture is altered with aging: trabecular thickness and numbers decrease, porosity increases, cortical bone mass is lost, and adiposity increases in the bone marrow. In humans, bone mass loss begins between 20 and 30 years in both men and women [13], with an acceleration in the perimenopause period.

The molecular and cellular bases underlying the pathogenesis of age-related bone changes are now well-known [14]. An increase in bone turnover and an imbalance in bone remodeling are observed in aging, mainly due to a reduction in OB differentiation and activity (reduced bone-forming capacity) (see [14] for the molecular bases of these phenomena). Both reduced OB differentiation and activity are the primary pathological mechanisms of low bone mass in the elderly and in animal models of aging. Moreover, an increase in osteoclastogenesis and osteoclast activity is also commonly observed, leading to less new bone formation and increased bone resorption.

The declines in differentiated cell function and stem cell reserves linked to senescence are important factors in age-related pathologies, and an increase in cellular senescence is particularly involved in bone aging [15,16]. Indeed, cellular senescence and the apoptosis of OB and osteocytes explain much of the bone aging phenotype. It also affects myeloid cells and osteoprogenitors. Many molecular and cellular factors are involved and have been reviewed elsewhere [15,16]. Importantly, age-related bone remodeling has effects on normal hematopoiesis because it affects the endosteal niche [17,18,19], which is a specialized microenvironment located in the bone marrow that plays a critical role in the regulation of hematopoietic stem cells (HSCs) [20,21]. The endosteal niche is located near the inner surface of the bone, where the presence of OB is thought to provide HSCs with signals that regulate their self-renewal and differentiation, ensuring that the bone marrow maintains a healthy and functional population of blood cells. This niche is also composed of a complex network of other cells, including endothelial cells, stromal cells, and OC. These cells produce a variety of signaling molecules, such as cytokines, growth factors, and extracellular matrix proteins that interact with HSCs and influence their behavior. Thus, it is a critical component of the bone marrow microenvironment (BME), and its proper function is essential for maintaining a healthy and functional hematopoietic system.

The bone area of the aging endosteal niche is characterized by a reduction in the number of OB and the release of osteopontin, which negatively regulates stem cell pool size [22]. Moreover, a degeneration of arteries and arterioles makes them less able to support HSC function [23], and adipocytes accumulate thanks to enhanced adipogenic differentiation of the mesenchymal stromal cells [24]. These phenomena lead to HSC differentiation skewed towards the myeloid lineages [25]. Thus, the skeletal bone aging clearly alters the BME. However, direct evidence of the direct correlations between the aged bone phenotype, niche alterations, and hematopoietic functions is still lacking [19].

MGUS is also characterized by associated bone changes that include (i) generalized bone loss, leading to bone fragility with increased fracture risk (reviewed in [5]), and (ii) a shift/disruption of the endosteal niche from an osteoblastic to an osteoclastic profile [26]. Of note, overt osteoporosis has been significantly associated with MGUS [5]. That is the reason why Drake suggests the term Monoclonal Gammopathy of Skeletal Significance (MGSS) rather than MGUS [5]. Of particular interest is the fact that generalized bone loss is also observed in MM with lytic bone lesions [4]. Thus, bone fragility/generalized bone loss is common to both MGUS and MM and present all along the evolution from MGUS to MM. Such bone changes could be the common context favoring the emergence of both MGUS and MM. Moreover, this shared bone fragility probably pre-exists in MGUS. Indeed, all recent works indicate that an END, in relation to bone senescence, including degeneration of the endosteal niche and bone inflammaging, is observed before MGUS development [17] and prefigures the END observed in MGUS and, subsequently, MM. The degree and rate of bone senescence, which is an early process in human life, can vary between individuals, possibly explaining the early occurrence of MGUS and MM in rare individuals (40–60 years old). Moreover, age-related changes in the immune response in both innate and adaptive immunity [27] parallels bone senescence timing and could be involved in the early appearance of MGUS and MM if both processes occur rapidly in some individuals.

## 3. Ins: Bone Inflammaging

The development of a chronic state of systemic, low-grade inflammation in the bone, defined as “sterile inflammation” and termed “inflammaging”, is another major component of bone aging [18]. As osteolineage cells age, they produce an overall inflammatory microenvironment and acquire the senescence-associated secretory phenotype (SASP) that corresponds to the secretion of pro-inflammatory molecules such as chemokines and cytokines, bioactive lipids, and exosomes [28]. Of note, in mice, the expression of the inflammatory cytokines IL1β and IL6, which have been reported to impair HSC self-renewal and enhance proliferation and myeloid bias, and of the inflammatory chemokines Cxcl2 and Cxcl5, is increased in the aged bone marrow stroma [29]. Thus, inflammaging is proposed to promote HSC aging and lymphoid to myeloid differentiation [30]. Indeed, aging-associated functional changes in HSCs such as reduced self-renewal and myeloid-skewed differentiation are very close to the ones that occur during inflammation [30]. This accumulation of myeloid cells that are prone to produce pro-inflammatory cytokines amplify the phenomenon. Moreover, the differentiation of mesenchymal stromal cells into adipocytes during aging also contributes to the SASP and enhanced myelopoiesis because adipocytes further secrete molecules that promote the proinflammatory immune phenotype, inhibit osteodifferentiation, and promote osteoclastogenesis [19].

Thus, the remodeling of bone marrow niches during aging leads to impaired or imbalanced skeletal homeostasis. As aging induces pro-inflammatory molecules, bone regeneration is impaired through decreased skeletal stem and progenitor cell numbers and osteogenic function [31,32]. Many studies suggest that inflammation influences the maturation and growth of osteocytes and OB [33]. Several studies have shown that in inflammatory conditions, both osteocytes [34] and OB [35,36] can produce pro-inflammatory cytokines and chemokines, thereby disrupting the homeostasis of the BME. Thus, they amplify local inflammation and generate an increase in bone resorption and a decrease in bone formation. Moreover, the role of inflammaging in regulating bone-resorbing OC was partly deciphered recently, when it was demonstrated that aged OC precursor macrophages accumulate the metabolite itaconate, with impacts to OC differentiation and bone homeostasis [37]. Thus, OC and macrophages belong to the same myeloid lineage, and itaconate plays a role in addition to the well-known OC differentiation and activation factors, including the ubiquitous GMCSF and the specific RANK ligand factors that are more abundant in the MM microenvironment [38]. More generally, the interplay between inflammation and pathological bone resorption is now well-characterized, with chronic inflammation causing a shift in the bone remodeling process toward pathological bone resorption [33].

Altogether, the inflammatory mediators produced in the SASP affect OC activity and inhibit OB differentiation, resulting in a reduction in bone formation, mineralization, and density. The END and generalized bone loss that are observed in MGUS/MGSS reflect an abnormal accentuation of this physiological process. The release of inflammatory cytokines observed in MGUS/MGSS beyond what is observed in normal individuals could explain such excesses of senescence [39]. Thus, excessive aging through endosteal niche degeneration and sterile bone inflammaging creates a permissive BME that favors MGUS/MGSS.

In summary, the END, characterized by a shift from an osteoblastic to an osteoclastic profile of the endosteal niche in relation to excessive bone senescence, could represent a permissive BME that facilitates not only the emergence of MM from MGUS [9,10,40] but also the emergence and maintenance of MGUS. Our purpose is now to envisage whether the appearance of MGUS and MM could originate from this age-related disrupted BME.

## 4. Outs: MGUS and MM Genesis According to TiDiS Theory

MM is formed of slow-cycling, non-fully differentiated plasma cells (PCs) that accumulate in the bone marrow. PCs correspond to the terminal stage of B-cell lineage differentiation. They secrete large amounts of antibodies and are the effectors of humoral immunity. In lymph nodes (germinal centers), secondary antigen presentation with T cells helps generate long-lived and highly mutated memory B cells and PCs, both of which are able to circulate and persist inside the bone marrow for many years. Memory B cells can be reactivated to enrich the pool of PCs. These highly mutated and long-lived PCs are the normal counterparts of MM cells [41].

**Figure 2:**
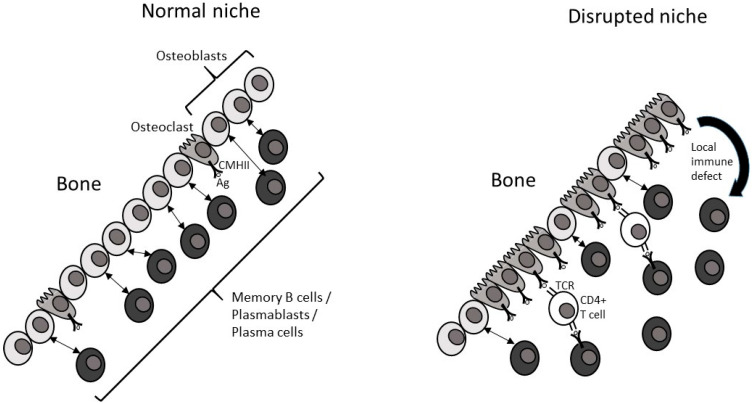
Hypothetical mechanisms for the consequences of endosteal niche disruption in the bone marrow. In the normal endosteal niche, all B cells and their derivatives, especially the plasma cells, are able to establish the required direct interactions with osteoblasts to fully differentiate in the context of polyclonal B cell activation (transient MGUS). Few osteoclasts present bone-derived antigens [46]. In the disrupted niche, due to bone senescence and inflammaging, the shift from an osteoblastic profile to an osteoclastic profile limits the possibility for plasma cells to interact with osteoblasts during polyclonal B cell activation. Thus, they cannot fully differentiate, and they stay in a state of instability with residual proliferation and increased stochasticity (epigenetic and genetic instability) that enhances the risk of MGUS stabilization. Moreover, the excess of osteoclasts favors the presentation of antigens into the niche and the expansion of the selected clones because they are immunosuppressive (local immune defect). Of note, other immune cells that do not appear in this figure are present in the MM tumor microenvironment, especially different subsets of macrophages (M1 or M2), among which the presence of M2-like tumor-associated macrophages is associated with poor prognoses [47,48].

Bone marrow niches in mice are known to facilitate the survival, maintenance, and differentiation of hematopoietic multipotent progenitors and PCs. Specific stromal cells and various factors in the niches are involved, and similarities between the microenvironments necessary for the establishment and the maintenance of these two immune cell subsets exist, including the CXCL12/CXCR4 signaling axis [8]. Although early stages of B lymphocyte differentiation requires important signals delivered by OB that are crucial cells for lymphocyte development [42], the idea that OB and endosteal niches control B-lymphopoiesis is currently challenged [43]. Nevertheless, it is clear that later, during immunopoiesis, immature PCs, known as plasmablasts or short-term PCs, formed in the secondary lymphoid organs then migrate to the BME where they terminate their maturation to become fully differentiated long-term PCs [8] (Figure 2). PC maintenance occurs in the BME thanks to the close contact established between stromal cells and PCs, especially through integrins and their ligands. Several factors of the bone marrow niches are essential for the maintenance of PCs, especially factors secreted by OB such as CXCL9 [8]. Recent syntheses suggested the existence of a multicellular niche for PCs within the BM, with several hematopoietic components and at least one stromal component that may correspond to osteoprogenitors. This niche could be shared with multipotent progenitors and HSCs and overlap with endosteal niches [8]. Of note, immature and fully differentiated PCs may have different locations depending on their maturation stages, but this precise question has never been assessed. In the MM context, MM cells seed the endosteal bone surface and interact with bone-lining cells, which induce MM cells to enter a dormant state [44,45].

MM-induced bone changes are the hallmark of MM. The mechanisms of MM-induced bone changes are specific to MM, suggesting that these bone changes could be more than simply induced and viewed as a fatal attraction regarding the interactions of MM cells for/with bones, as it could also be primarily involved in the oncogenesis of MM from MGUS. Although such lytic bone lesions result from a disruption of the bone remodeling compartment, which is characterized by an uncoupling between an excess of bone resorption and a suppression of bone formation, we were the first to show that the major factor responsible for such lytic bone lesions was the suppression of bone formation, which is a disruption of the mesenchymal stromal to OB transition [3]. Of note, this OB suppression with marked osteoblastic senescence is unique and specific to MM (when compared to what is observed in bone metastases) [49]. For this reason, we hypothesized that because the mechanisms of lytic bone lesions were unique and specific, the BME could play a major role in MM oncogenesis, meaning that bone could be more than ‘a fatal attraction and interaction’ in MM and could be causal. Thus, we have been in search of a unique and specific bone scenario for the emergence of MM from MGUS.

We previously proposed that age-related disruption of the BME and endosteal niches would increase the pool of PCs that cannot niche into the BME during polyclonal B cell activation and transient MGUS, when the number of PCs needing to fully differentiate is enhanced [10,40]. As a consequence, more PCs would remain in an unstable phenotypic state during this phase of transient MGUS, with an increased risk of acquiring genetic alterations [9,10,40] (Figure 2). The subsequent increased cellular stochasticity linked to the inability to interact with the required cellular and molecular partners in the niche would be the first step towards a possible transformation in MM, according to this TiDiS theory. If a genomic alteration preexists in a given clone, the risk of MGUS stabilization would be increased, and the oncogenic process into MM could be accelerated, but it does not mean that the process cannot be initiated without genomic alterations. The sole END can be considered as the initial step in this bone scenario. Moreover, the presence of oncogenic alterations alone is not a sufficient factor to produce MM [9,10,40], showing the importance of a permissive BME context, whereas these bone changes have been systematically observed in both stabilized MGUS and MM.

In summary, an early (pre-MGUS) END, in relation to increased bone senescence (endosteal niche degeneration and bone inflammaging), prefigures the disruption of the bone remodeling compartment observed first in MGUS/MGSS and then in MM. We propose (Figure 2) that the END, more precisely the shift from the OB to OC profile (excess of OC), could play a major role in (i) favoring the presentation of antigens (Ag) into the endosteal niche because osteoclasts are Ag-presenting cells [46], (ii) favoring the expansion of such selected clones because OC are immunosuppressive (local immune defect), and (iii) favoring stochasticity (epigenetic and genetic instability) in selected clones as previously discussed in the TiDiS theory [10,40]. Thus, END could impact/favor the presentation of some specific Ag inside the bone marrow, select specific clones, leading to clonal expansion/MGUS, and increase the stochasticity inside such selected immune clones, facilitating their malignant transformation into MM. Finally, beyond the well-characterized role of many immune cells in the MM microenvironment, especially subsets of macrophages and T cells [47,48], we propose consideration of a new major partner, which is at the interface between the bone and the immune compartments.

## 5. Outs: Immune Effects, Lessons from Gaucher Disease

Until now, infection, inflammation, and immune deficiency have been the most documented contexts for the emergence of MGUS. In these contexts, the role of Ag appears to be of major importance. Many studies have now demonstrated the presence of immunoglobulin (Ig)-gene mutations in both MGUS and MM, showing that both MGUS and MM clones are clones engaged in an immune response [1]. Moreover, the Ag specificity of these clones has been re-evaluated, and this specificity was determined in more than 60% of monoclonal Ig [50]. Of note, whereas the Ig-gene mutations of MM clones are fixed, showing that MM is a process independent of Ag, Ig-gene mutations in MGUS are ongoing, showing that the MGUS clones remain dependent on Ag [51]. This is a major argument in favor of operational Ag activation in sporadic MGUS because the machinery of Ig-gene mutations remains operational in MGUS, whereas mutations are definitely fixed in MM.

Then, the central question is: can the bone marrow present Ag by itself [52]? It is indeed the case because OC are themselves Ag-presenting cells, as macrophages and dendritic cells are, and bone marrow is thus an Ag-presenting site, as germinal centers are [46] (Figure 2). However, is there specificity for some Ag, and are the Ag recognized in MGUS any Ag, or are they specific to the bone marrow? An important element of response is that they are often Ag (auto-Ag) linked to human metabolism (and not to bacterial or viral infections).

From our model, we propose that END can favor the presentation of these specific Ag by the OC (Figure 2). Indeed, the disruption could enhance their presentation because the niche becomes osteoclastic. Either the disruption creates the Ag presentation, or, most probably, it strongly increases it. The sequence of events would be that senescence/infection/inflammaging creates an increase in Ag presentation, producing a permanent and strong antigenic stimulation. Thus, OC could have a selective role in the selection of specific clones (or in the limitation of the response, which would limit the expansion of a given clone).

Recent elegant studies in patients with GD, in whom an excess of both MGUS and MM was observed, are highly relevant to this concept. GD is characterized by an increased incidence of hyper-gammaglobulinemia (100%), MGUS (30%), and MM (4%) with this characteristic sequence: hyper-gammaglobulinemia, then MGUS, then MM [12]. Thus, GD recapitulates the natural history of MM from MGUS via polyclonal B cell activation. Moreover, there is also a GD bone disease characterized by a disruption of the bone remodeling compartment (END) [53]. In GD, sphingolipids accumulate inside the bone marrow because of an enzyme deficiency. Thus, one Ag (sphingolipid) is massively present in GD, especially in the bone marrow. Of note, GD-associated monoclonal immunoglobulins (in case of MGUS or MM) specifically recognize sphingolipids as their Ag [54,55,56]. This suggests permanent/prolonged presentation and activation of this Ag inside the bone marrow. Thus, MGUS emerges with antibodies specific to this Ag. GD-associated MGUS recognizes sphingolipids, the antigen specifically involved in the pathogenesis of GD, which accumulates inside the bone marrow.

Strikingly, this antigenic specificity for the sphingolipids of GD-associated monoclonal immunoglobulins is also observed in 15–20% of sporadic MGUS [54], suggesting that the GD model can be generalized. Thus, there is probably a permanent activation from specific Ag that favors the emergence of permanent MGUS. Ig-gene mutations remain active in sporadic MGUS, suggesting that MGUS remains dependent on a specific antigen, which is frequently (but not always) an auto-antigen present inside the bone marrow. Finally, MGUS is suggested to emerge from an inflammatory environment in a manner similar to Ig/auto-antibodies in auto-immune diseases [39]. Thus, permanent MGUS cases are probably due to the permanent stimulation by an Ag in the whole organism, especially the bone marrow.

## 6. Conclusions

Inside the bone marrow, now considered as an antigen-presenting site (at least for certain antigens as auto-antigens), our comprehensive bone scenario for both MGUS and MM initiates with bone senescence and bone inflammaging (END) that has three consequences: (i) abnormal Ag presentation through OC as Ag-presenting cells in excess, (ii) local immune defects through OC, and (iii) increased stochasticity (epigenetic and genetic instability) in selected clones. END, through the activation of OC as Ag-presenting cells, could significantly stimulate the presentation of some specific Ag and the clonal expansion of such stimulated cells. The presence of such Ag inside the bone marrow could also facilitate the maintenance of such selected clones. This scenario that integrates bone senescence, immune stimulation, the BME, and the genesis of MGUS and MM provides a new perspective at the tissue level that appears to better reconcile the different sides of the disease than canonical, genome-centered perspectives.

## Data Availability

Not applicable.
